# Early Tonsillectomy for Dual Remission of Periodic Fever, Aphthous Stomatitis, Pharyngitis, and Adenitis Syndrome and Immunoglobulin A Nephropathy: A Case Report

**DOI:** 10.1016/j.xkme.2026.101377

**Published:** 2026-04-27

**Authors:** Daishi Hirano, Kensuke Joh, Shiko Honma, Ryoko Sakaguchi, Aya Saito, Yuna Hiwatari, Shiho Yoshizawa, Yosuke Shoji, Haruhide Sakaguchi, Chisato Umeda, Saori Miwa, Akira Ito

**Affiliations:** 1 Department of Pediatrics, The Jikei University School of Medicine, Tokyo, Japan; 2 Department of Pathology, The Jikei University School of Medicine, Tokyo, Japan; 3 Division of Nephrology, Saitama Children’s Medical Center, Saitama, Japan

**Keywords:** Tonsillectomy, PFAPA syndrome, IgA nephropathy, simultaneous remission, pediatric autoinflammatory disease, focal infection

## Abstract

Despite sharing tonsillar immune dysregulation as a common pathogenic mechanism, concurrent presentation of periodic fever, aphthous stomatitis, pharyngitis, and adenitis (PFAPA) syndrome with immunoglobulin A (IgA) nephropathy (IgAN) remains exceptionally rare, with only 1 previous case reported in the literature receiving multidrug immunosuppressive therapy before tonsillectomy. In this study, we report the first pediatric case in which both conditions achieved sustained remission following early tonsillectomy without additional immunosuppression. A 6-year-old boy with a 3-year history of PFAPA developed gross hematuria coinciding with febrile episodes. Kidney biopsy confirmed IgAN. Owing to the poor response to cimetidine and significant disruption to schooling, palatine tonsillectomy was performed at 7 years. Following surgery, PFAPA episodes completely resolved, and hematuria ceased. Repeat kidney biopsy at 11 months postoperatively showed marked histological improvement, with resolution of mesangial hypercellularity and reduced endocapillary proliferation. This case provides the first pediatric case demonstrating that early tonsillectomy alone can effectively treat coexisting PFAPA and IgAN without the need for additional immunosuppressive therapy. The simultaneous clinical and histological remission supports a shared pathogenic mechanism involving tonsillar immune dysfunction and highlights tonsillectomy as a potentially curative intervention for select patients with concurrent tonsil-driven diseases.

## Introduction

Periodic fever, aphthous stomatitis, pharyngitis, and adenitis (PFAPA) syndrome and immunoglobulin A (IgA) nephropathy (IgAN) are systemic diseases in which tonsillar dysfunction is thought to act as a local immune trigger. Both share pathogenic mechanisms involving aberrant tonsillar immune regulation.^1^, ^2^, ^3^, ^4^, ^5^ PFAPA syndrome, a pediatric autoinflammatory disorder characterized by recurrent febrile episodes, is associated with dysregulated interleukin 1 signaling and chronic tonsillar inflammation.^4^^,^^6^, ^7^, ^8^ IgAN, the most common form of primary glomerulonephritis, has a multihit pathogenesis involving galactose-deficient IgA1, antiglycan autoantibodies, immune complex formation, and complement-mediated glomerular injury.^9^ Tonsillar mucosal immune responses are implicated in a subset of patients, supporting a shared immunological origin. Despite these overlaps, concurrent PFAPA and IgAN is rare, with only 1 case reported to date.^10^

Both conditions are responsive to tonsillectomy. A meta-analysis of PFAPA syndrome reported a complete resolution rate of febrile episodes in ∼83% (95% confidence interval, 77%-89%),^11^ whereas multicenter studies of IgAN have shown reduced proteinuria and hematuria and improved histological outcomes with tonsillectomy combined with steroid pulse therapy.^12^

We report the first pediatric case of concurrent PFAPA and IgAN achieving complete remission of both diseases with tonsillectomy monotherapy, offering further insights into the shared immunopathogenesis and supporting tonsillar immune dysfunction as a unifying therapeutic target.

## Case Report

A 6-year-old boy presented with gross hematuria and proteinuria coinciding with febrile episodes. Since the age of 3 years, he had experienced recurrent fevers exceeding 38 °C, lasting 3-5 days every 2-4 weeks. Episodes were characterized by tonsillar swelling with white exudate in the absence of upper respiratory symptoms. He remained completely asymptomatic between episodes and exhibited normal growth and development. At the age of 4 years and 4 months, he was diagnosed with PFAPA syndrome based on clinical diagnostic criteria described by Thomas et al,^13^ fulfilling all requirements including periodic fevers, exudative tonsillitis, cervical lymphadenitis, and asymptomatic intervals. Laboratory findings during febrile episodes showed marked systemic inflammation as follows: white blood cell count 16,090/μL (neutrophils 78.4%), C-reactive protein 7.44 mg/dL, and serum amyloid A 897 mg/dL. Conversely, procalcitonin was notably low (0.06 ng/mL), suggesting autoinflammatory flares rather than bacterial infection. All inflammatory markers completely normalized during afebrile periods. Prophylactic cimetidine (15 mg/kg per d in 2 divided doses) was initiated but showed limited efficacy, with febrile episodes continuing at 1-month intervals.

At the age of 6 years and 9 months, gross hematuria and proteinuria (4+) developed during a febrile episode, with a urinary protein-creatinine ratio of 3.13 g/gCr, which recurred 2 weeks later. He exhibited recurrent gross hematuria coinciding with each PFAPA episode, leading to school absences and reduced family quality of life.

Between febrile episodes, urinalysis showed resolution of proteinuria (urinary protein-creatinine ratio 0.12 g/gCr), but persistent microscopic hematuria (3+ occult blood) and glomerular-type dysmorphic red blood cells (30%), suggesting ongoing glomerular pathology. Family history was positive only for paternal asymptomatic hematuria. Although isolated asymptomatic microscopic hematuria usually does not warrant kidney biopsy in children, a biopsy was performed after family discussion because of recurrent gross hematuria temporally associated with PFAPA flares and frequent school absences.

At biopsy (age 6 years and 11 months, asymptomatic), growth parameters and blood pressure were normal. Physical examination revealed bilateral palatine tonsillar hypertrophy (Mackenzie grade 2/3) without erythema or exudate. Laboratory results including renal function, complements, and autoimmune markers were normal.

**Figure 1:**
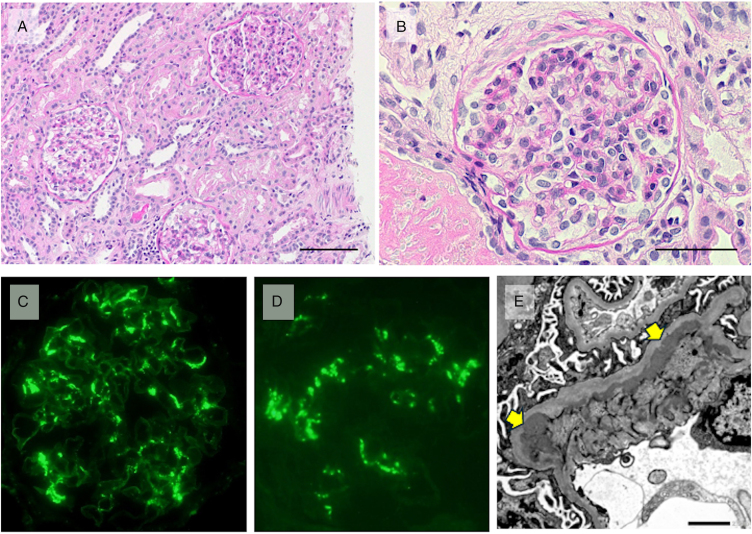
Initial kidney biopsy findings at the age of 6 years and 11 months. Histological evaluation revealed diffusely and mildly hypercellular glomeruli. Mesangial hypercellularity was present in 7 glomeruli (13%), endocapillary hypercellularity in 1 glomerulus (2%), and a fibrous crescent in 1 glomerulus (2%). No active crescents, global or segmental sclerosis, tubular atrophy, or interstitial fibrosis was observed (panel A, periodic acid–Schiff stain; scale bar: 100 μm). A representative glomerulus showed mesangial and endocapillary hypercellularity along with a fibrous crescent (panel B, periodic acid–Schiff stain; scale bar: 50 μm). Immunofluorescence demonstrated granular immunoglobulin A (IgA) and C3c deposition in the mesangium (panel C, IgA; panel D, C3c). Electron microscopy revealed mesangial electron-dense deposits (panel E, scale bar: 2.0 μm). The pathological diagnosis was IgA nephropathy (Oxford classification: M1S0E1T0C0; H grade I).

Kidney biopsy revealed 55 glomeruli with mesangial hypercellularity in 7 glomeruli (13%) and endocapillary hypercellularity and fibrous crescent in 1 glomerulus each (2%). Immunofluorescence demonstrated mesangial IgA and C3c deposition, and electron microscopy revealed mesangial electron-dense deposits. A diagnosis of IgAN (M1S0E1T0C0) was made (Fig 1).

Given the poor response to cimetidine and the burden of frequent febrile episodes on the patient’s quality of life and education, palatine tonsillectomy was performed at the age of 7 years and 2 months. Postoperatively, PFAPA episodes ceased completely, and cimetidine was discontinued.

**Figure 2:**
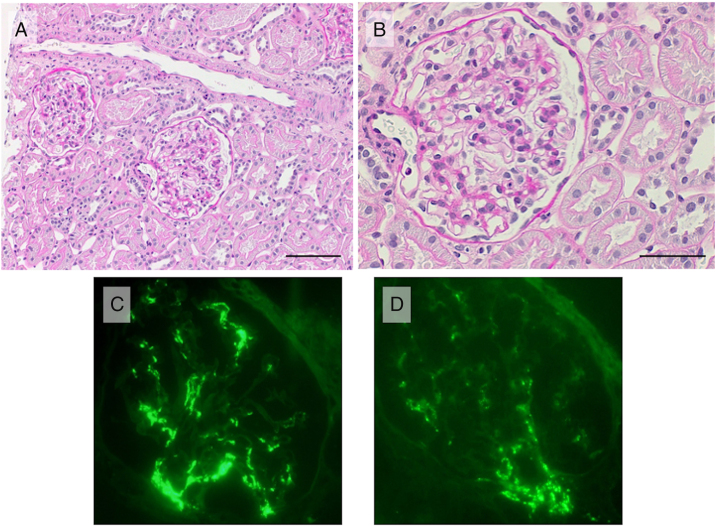
Follow-up kidney biopsy findings at 11 months posttonsillectomy. Follow-up biopsy at 11 months posttonsillectomy showed marked histological improvement, with no mesangial hypercellularity, endocapillary hypercellularity, or crescent formation (panel A, periodic acid–Schiff stain; scale bar: 100 μm). A representative glomerulus showed normal mesangium and minimal endocapillary changes (panel B, periodic acid–Schiff stain; scale bar: 50 μm). Immunofluorescence revealed persistent granular immunoglobulin A and C3c deposition, although the intensity of C3c was reduced compared with the initial biopsy (panel C, immunoglobulin A; panel D, C3c). These findings confirmed substantial improvement in glomerular inflammation following tonsillectomy.

Remarkably, hematuria resolved completely within 1 month after surgery, and the absence of PFAPA febrile episodes eliminated the previously consistent pattern of episodic gross hematuria. Follow-up kidney biopsy at 11 months posttonsillectomy demonstrated marked histological improvement consistent with Oxford classification regression. Although immunofluorescence continued to show mesangial IgA and C3c deposition, C3c staining was reduced compared with the initial biopsy. The updated Oxford classification was M0S0E0T0C0 (Fig 2).

**Figure 3:**
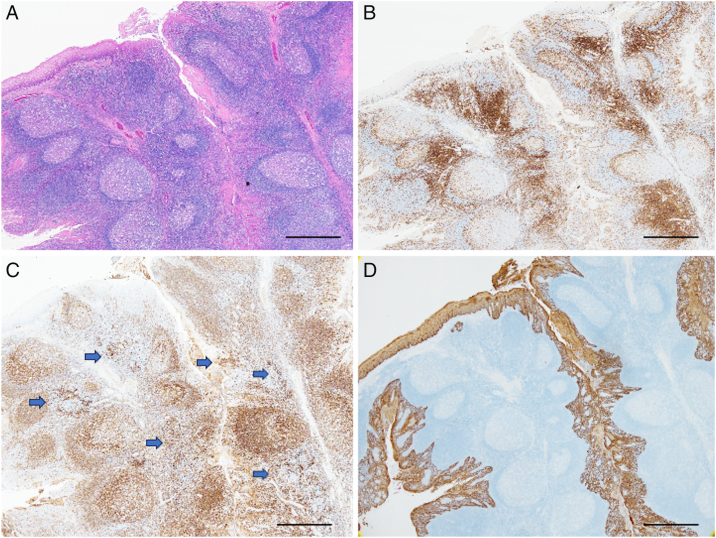
Histological and immunohistochemical findings of the palatine tonsil. Representative sections of the resected palatine tonsil stained with hematoxylin and eosin (A), anti-CD4 (B), anti-HLA-DP, -DQ, and -DR (C), and anticytokeratin AE1/AE3 (D). Scale bar: 500 μm. (A) The tonsillar architecture revealed regularly shaped lymphoid follicles with well-developed germinal centers. The mantle zone appeared as an elliptical bulge directed toward the crypts. (B) CD4-positive cells were distributed prominently in both extrafollicular areas and within germinal centers, with notable staining on the dorsal side of lymphoid follicles. (C) Clusters of HLA-DP--, HLA-DQ--, and HLA-DR--positive dendritic cells were observed within the CD4-positive T-nodule region (arrows). (D) The tonsillar crypts exhibited preserved lymphoepithelial symbiosis, a network-like structure in which epithelial cells and lymphocytes coexist. Anticytokeratin AE1/AE3 staining revealed a characteristic loose reticular pattern of this lymphoepithelial arrangement.

Immunohistochemical analysis of resected tonsils revealed expansion of CD4-positive regions and clustering of dendritic cells positive for HLA-DP, -DQ, and -DR within these same areas. These findings were more prominent than those typically seen in age-matched patients with chronic tonsillitis,^14^ indicating an aberrant immunological profile. Furthermore, lymphoepithelial symbiosis within the tonsillar crypt epithelium was preserved, suggesting that the tonsils had been in a state of active inflammation consistent with IgAN-associated tonsillitis (Fig 3).

The patient has remained free of PFAPA recurrence, with normal urinalysis results over the 2-year follow-up period.

## Immunohistochemical Examination

Tonsillar sections were stained using the VENTANA BenchMark ULTRA automated system (Ventana Medical Systems) with Cell Conditioning 1 buffer (Ventana Medical Systems) for 36 minutes. Primary antibodies used were mouse monoclonal anti-HLA-DP, -DQ, and -DR; anti-CD4; and anticytokeratin (AE1/AE3).

## Discussion

This case provided evidence for shared pathogenic mechanisms between PFAPA syndrome and IgAN, both involving dysregulated mucosal immunity centered on tonsillar dysfunction.^1^, ^2^, ^3^, ^4^, ^5^ Recent molecular studies identified specific tonsillar abnormalities in PFAPA syndrome, including persistent nuclear factor κB activation, sustained interleukin 1β pathway priming, and increased infiltration of CD4^+^/CD8^+^ T cells and memory B cells, even during asymptomatic periods, indicating a persistent proinflammatory tonsillar milieu that predisposes to systemic immune activation.^6^, ^7^, ^8^

In IgAN, emerging evidence supports the “tonsil-kidney axis” hypothesis, by which aberrant mucosal immunity drives the 4-hit pathogenesis, including overproduction of galactose-deficient IgA1, formation of antiglycan autoantibodies, immune complex deposition, and complement-mediated glomerular injury.^9^^,^^15^^,^^16^ Recent studies of tonsillar disorders demonstrated increased interleukin 6, tumor necrosis factor α, and interferon γ expression,^6^^,^^17^ promoting B-cell activation and abnormal IgA class switching, potentially linking both conditions in our patient.^16^^,^^18^

Cell-mediated immunity also plays a critical role in IgAN pathogenesis. Tonsillar tissue from IgAN patients shows distinct histological features, including accelerated germinal center regression, T-nodule formation, and altered lymphoepithelial symbiosis, correlated with chronic glomerular injury.^2^^,^^3^^,^^14^ Although lymphoepithelial symbiosis is typically poorly developed in IgAN patients with long-standing disease, it remained well preserved in our patient. This may be explained by early tonsillectomy at 7 years of age and the unique episodic nature of PFAPA. In our case, the repeated acute inflammatory stimuli from PFAPA may have maintained the tonsils in a state of active mucosal immune response, preventing the structural regression often observed in chronic, indolent IgAN tonsillitis. Furthermore, tonsillectomy has shown clinical efficacy in other tonsillar focal diseases, such as palmoplantar pustulosis, supporting the broader role of tonsillar immune dysfunction in systemic manifestations. In particular, differentiated memory T cells, specifically CD8^+^/CX3CR1^+^ cells, migrate to glomeruli and promote inflammatory cell recruitment via the CX3CR1-fractalkine axis.^2^^,^^19^

Dramatic clinical and histological improvement following tonsillectomy alone in our case suggests that removing the primary source of aberrant immune activation effectively interrupted the pathogenic cascade for both conditions. Complete cessation of PFAPA episodes and resolution of gross hematuria, along with reduction in mesangial hypercellularity from 13%-0% within 11 months, support the dual therapeutic benefit of tonsillectomy. Importantly, persistent IgA deposition on immunofluorescence despite clinical and histological remission suggests that tonsillectomy acts by halting ongoing immune activation rather than clearing pre-existing immune complexes, consistent with previous studies showing dissociation between immune deposition and disease activity in IgAN.^5^

Although IgAN severity was relatively mild, the following factors justified surgical intervention: (1) progressively shortened PFAPA intervals that caused significant educational disruption, (2) recurrent gross hematuria suggestive of active glomerular inflammation, (3) poor response to cimetidine, and (4) the opportunity to address both conditions with a single intervention.^4^

Although this case demonstrated remarkable efficacy of tonsillectomy monotherapy, several limitations must be acknowledged. The possibility of spontaneous remission cannot be excluded; the strong temporal association between tonsillectomy and clinical/histological improvement supported its therapeutic role. Longer term follow-up beyond 2 years will be necessary to confirm durable remission.

Our case differed from the only previous report of concurrent PFAPA and IgAN by Sugimoto et al,^10^ in which the patient received extensive immunosuppressive therapy including corticosteroids, immunosuppressants, anticoagulants, and angiotensin-converting enzyme inhibitors before tonsillectomy. In contrast, our patient achieved complete remission with tonsillectomy as the primary intervention, potentially sparing pediatric patients the adverse effects of long-term immunosuppression. The histological and immunological findings in resected tonsils, such as the expansion of T nodules and clustering of mature dendritic cells, were consistent with those reported in previous studies of PFAPA syndrome without renal involvement.^14^ This suggests that the intense, episodic immune activation inherent to PFAPA may serve as a potent trigger for the "tonsil-kidney axis" in certain patients. Whether additional specific factors are required for the development of IgAN in PFAPA patients remains to be elucidated using comparative analyses.

Future studies are needed to identify predictive biomarkers, such as serial serum IgA and galactose-deficient IgA1 measurements, and to clarify long-term renal outcomes following tonsillectomy in pediatric IgAN.^1^^,^^2^^,^^6^ Prospective studies examining long-term renal outcomes following tonsillectomy in pediatric IgAN are needed to establish evidence-based treatment guidelines.^1^^,^^5^

This case establishes tonsillectomy as a potentially curative, single-modality intervention for selected patients with concurrent PFAPA and IgAN. It underscores the therapeutic importance of targeting a shared immunological source and reinforces the role of tonsillar immune dysfunction as a central contributor to both conditions.^20^

## References

[1] Nakayama T., Kaneko H., Suzuki Y. (2024). Chronic tonsillitis and IgA nephropathy: findings from a nationwide Japanese cohort study. Am J Kidney Dis.

[2] Yamaguchi H., Goto S., Takahashi N. (2021). Aberrant mucosal immunoreaction to tonsillar microbiota in immunoglobulin A nephropathy. Nephrol Dial Transplant.

[3] Hotta O., Ieiri N., Nagai M., Tanaka A., Harabuchi Y. (2022). Role of palatine tonsil and epipharyngeal lymphoid tissue in the development of glomerular active lesions (glomerular vasculitis) in immunoglobulin A nephropathy. Int J Mol Sci.

[4] Manthiram K., Lapidus S., Edwards K. (2017). Unraveling the pathogenesis of periodic fever, aphthous stomatitis, pharyngitis, and cervical adenitis through genetic, immunologic, and microbiologic discoveries: an update. Curr Opin Rheumatol.

[5] Xie Y., Chen X., Nishi S., Narita I., Gejyo F. (2004). Relationship between tonsils and IgA nephropathy as well as indications of tonsillectomy. Kidney Int.

[6] Luu I., Sharma A., Guaderrama M. (2020). Immune dysregulation in the tonsillar microenvironment of periodic fever, aphthous stomatitis, pharyngitis, adenitis (PFAPA) syndrome. J Clin Immunol.

[7] Türkuçar S., Bülbül G., Ünsal E. (2023). Exploring the immunological basis of periodic fever, aphthous stomatitis, pharyngitis, and adenitis (PFAPA) syndrome: immunohistochemical staining features of palatine tonsils. Clin Rheumatol.

[8] Anselmi F., Dusser P., Kone-Paut I. (2025). Periodic fever, aphthous stomatitis, pharyngitis, and cervical adenitis (PFAPA) syndrome in children-from pathogenesis to treatment strategies: a comprehensive review. Paediatr Drugs.

[9] Cheung C.K., Alexander S., Reich H.N., Selvaskandan H., Zhang H., Barratt J. (2025). The pathogenesis of IgA nephropathy and implications for treatment. Nat Rev Nephrol.

[10] Sugimoto K., Fujita S., Miyazawa T., Okada M., Takemura T. (2013). Periodic fever, aphthous stomatitis, pharyngitis, and adenitis (PFAPA) syndrome and IgA nephropathy. Pediatr Nephrol.

[11] Garavello W., Pignataro L., Gaini L., Torretta S., Somigliana E., Gaini R. (2011). Tonsillectomy in children with periodic fever, aphthous stomatitis, pharyngitis, and adenitis syndrome. J Pediatr.

[12] Kawamura T., Yoshimura M., Miyazaki Y. (2014). A multicenter randomized controlled trial of tonsillectomy combined with steroid pulse therapy in patients with immunoglobulin A nephropathy. Nephrol Dial Transplant.

[13] Thomas K.T., Feder H.M., Lawton A.R., Edwards K.M. (1999). Periodic fever syndrome in children. J Pediatr.

[14] Joh K., Ueda H., Katayama K., Kitamura H., Watanabe K., Hotta O. (2024). Histological correlation between tonsillar and glomerular lesions in patients with IgA nephropathy justifying tonsillectomy: a retrospective cohort study. Int J Mol Sci.

[15] Suzuki H., Kiryluk K., Novak J. (2011). The pathophysiology of IgA nephropathy. J Am Soc Nephrol.

[16] Placzek W.J., Yanagawa H., Makita Y. (2018). Serum galactose-deficient-IgA1 and IgG autoantibodies correlate in patients with IgA nephropathy. PLoS One.

[17] Stojanov S., Lapidus S., Chitkara P. (2011). Periodic fever, aphthous stomatitis, pharyngitis, and adenitis (PFAPA) is a disorder of innate immunity and Th1 activation responsive to IL-1 blockade. Proc Natl Acad Sci U S A.

[18] Du W., Gao C.Y., You X. (2022). Increased proportion of follicular helper T cells is associated with B cell activation and disease severity in IgA nephropathy. Front Immunol.

[19] Audemard-Verger A., Pillebout E., Jamin A. (2019). Recruitment of CXCR3+ T cells into injured tissues in adult IgA vasculitis patients correlates with disease activity. J Autoimmun.

[20] Theodoropoulou K., Vanoni F., Hofer M. (2016). Periodic fever, aphthous stomatitis, pharyngitis, and cervical adenitis (PFAPA) syndrome: a review of the pathogenesis. Curr Rheumatol Rep.

